# The Intervening Sequence of *Coxiella burnetii*: Characterization and Evolution

**DOI:** 10.3389/fcimb.2016.00083

**Published:** 2016-08-19

**Authors:** Indu Warrier, Mathias C. Walter, Dimitrios Frangoulidis, Rahul Raghavan, Linda D. Hicks, Michael F. Minnick

**Affiliations:** ^1^Program in Cellular, Molecular and Microbial Biology, Division of Biological Sciences, University of MontanaMissoula, MT, USA; ^2^Bundeswehr Institute of MicrobiologyMunich, Germany; ^3^Department of Biology and Center for Life in Extreme Environments, Portland State UniversityPortland, OR, USA

**Keywords:** intervening sequence, Coxiella, RNA, IVS, S23 protein

## Abstract

The intervening sequence (IVS) of *Coxiella burnetii*, the agent of Q fever, is a 428-nt selfish genetic element located in helix 45 of the precursor 23S rRNA. The IVS element, in turn, contains an ORF that encodes a hypothetical ribosomal S23 protein (S23p). Although S23p can be synthesized *in vitro* in the presence of an engineered *E. coli* promoter and ribosome binding site, results suggest that the protein is not synthesized *in vivo*. In spite of a high degree of IVS conservation among different strains of *C. burnetii*, the region immediately upstream of the S23p start codon is prone to change, and the S23p-encoding ORF is evidently undergoing reductive evolution. We determined that IVS excision from 23S rRNA was mediated by RNase III, and IVS RNA was rapidly degraded, thereafter. Levels of the resulting 23S rRNA fragments that flank the IVS, F1 (~1.2 kb) and F2 (~1.7 kb), were quantified over *C. burnetii*'s logarithmic growth phase (1–5 d). Results showed that 23S F1 quantities were consistently higher than those of F2 and 16S rRNA. The disparity between levels of the two 23S rRNA fragments following excision of IVS is an interesting phenomenon of unknown significance. Based upon phylogenetic analyses, IVS was acquired through horizontal transfer after *C. burnetii*'s divergence from an ancestral bacterium and has been subsequently maintained by vertical transfer. The widespread occurrence, maintenance and conservation of the IVS in *C. burnetii* imply that it plays an adaptive role or has a neutral effect on fitness.

## Introduction

*Coxiella burnetii*, the etiological agent of Q (query) fever in humans, is an obligate intracellular pathogen that replicates in a parasitophorous vacuole (PV; Voth and Heinzen, 2007). Q fever is a zoonotic disease that is generally acquired through inhalation of contaminated aerosols and is characterized by a flu-like illness accompanied by pneumonia and hepatitis (Maurin and Raoult, 1999). In less than 5% of cases, a chronic form with endocarditis as the predominant clinical symptom can occur. The bacterium undergoes a biphasic developmental cycle consisting of two cellular forms, including an infectious, dormant, small-cell variant (SCV), and a metabolically-active, large-cell variant (LCV). In spite of its intracellular nature, where chances for horizontal gene transfer are minimum, the genome of *C. burnetii* contains 31 insertion sequence (IS) elements spread across its genome, an intein in the C-terminal region of the replicative DNA helicase (DnaB) and two self-splicing group I introns (Cbu_L1917 and Cbu_L1951) along with an IVS in the 23S rRNA gene (Minnick and Raghavan, 2011).

IVSs are parasitic genetic elements that are found in 16S and 23S rRNA genes and are transcribed as part of the rRNA precursor. Excision of IVS elements occurs post-transcriptionally without ligation of the resulting fragments, which are held together by secondary and tertiary structures, thereby maintaining functional integrity of the 50S ribosome (Ralph and McClelland, 1993). IVSs have been identified in several bacteria, including *Salmonella* (Burgin et al., 1990), *Leptospira* (Ralph and McClelland, 1993), *Yersinia* (Skurnik and Toivanen, 1991), *Campylobacter* (Konkel et al., 1994), *Proteus* and *Providencia* (Miller et al., 2000). Within these bacteria, IVSs occur only in certain isolates, and not all *rrn* operons contain the IVS in a given bacterial strain (Burgin et al., 1990; Hsu et al., 1992; Mattatall and Sanderson, 1996; Miller et al., 2000). This sporadic distribution of IVS suggests the possibility of horizontal transfer of the element among eubacteria. Although the distribution of IVSs is random, their location within the 23S rRNA is relatively well-conserved. The most common sites for IVS insertion include counterparts of helices 9, 25, and 45 of the secondary structure of *E. coli*'s 23S rRNA (Noller, 1984). IVSs in both helix 25 and 45 are observed in *Salmonella* (Pabbaraju et al., 2000), *Helicobacter* (Hurtado et al., 1997), *Proteus* and *Providencia* (Miller et al., 2000). On the other hand, a single copy of IVS is found in helix 45 of *Leptospira* (Ralph and McClelland, 1993), *Yersinia* (Skurnik and Toivanen, 1991), *Campylobacter* (Konkel et al., 1994), and in helix 9 of various Alphaproteobacteria (Evguenieva-Hackenberg and Klug, 2000). Each of these helices originally consisted of a small tetraloop that was replaced by the extended stem-loop structure of an IVS. Conservation of IVS insertion sites across species indicates that continuity of the 23S rRNA at these positions is not necessary for ribosome function.

The IVS of *C. burnetii* was originally identified as a 444-nt element inserted in the single-copy 23S rRNA gene (Afseth et al., 1995). The element is bordered by complementary sequences that can form a stable stem-loop structure. IVSs of *Salmonella, Leptospira*, and many Alphaproteobacteria are known to be cleaved from their respective 23S rRNA by RNase III (Burgin et al., 1990; Ralph and McClelland, 1993; Evguenieva-Hackenberg and Klug, 2000), an endoribonuclease that cleaves pre-23S and pre-16S rRNA from a 30S rRNA precursor during maturation (Deutscher, 2009). Therefore, we initially hypothesized that *Coxiella*'s IVS was also excised from its 23S rRNA precursor by RNase III. Additionally, the IVS of *C. burnetii* contains an ORF that potentially encodes a ribosomal S23p family of hypothetical proteins. Although these proteins have no known function (Minnick and Raghavan, 2011), the crystal structure of the S23p of *Xanthomonas campestris* has been solved and found to be a homopentamer comprised of four-helix bundles creating a toroidal structure (Lin et al., 2006). In addition to *Xanthomonas*, S23p orthologs are also encoded by IVS ORFs of *Leptospira* and *Brucella* species. However, the only previous report on *C. burnetii*'s IVS suggested that translation of S23p may be inefficient since a poor Shine-Dalgarno (SD) sequence occurs upstream of the start codon of the respective ORF (Afseth et al., 1995).

At this point, very little is known about the biological role of IVSs in bacteria. The occurrence of IVSs across many bacterial species and its conservation in all strains of *C. burnetii* suggest that it could play an adaptive role. Interestingly, IVS elements appear to be most common in bacteria that form close associations with eukaryotes, where they are hypothesized to promote communication between bacteria and their hosts (Baker et al., 2003). Such a role is conceivable in *Coxiella* in view of its intracellular niche. A second possible role for IVS is in regulating pathogenesis. This possibility is supported by an observation that all pathogenic strains of *Y. enterocolitica* were found to possess an IVS, while non-pathogenic strains did not (Skurnik and Toivanen, 1991). A third possible role for IVS is in regulating rRNA degradation during *C. burnetii*'s development cycle. Studies in *Salmonella* have shown that the rate of 23S rRNA degradation during stationary phase is directly related to the degree of IVS-mediated fragmentation of the 23S rRNA (Hsu et al., 1994). Since *C. burnetii* transitions to a spore-like SCV near the end of its life-cycle, this adaptation could be advantageous.

The aim of this study was to more fully characterize the IVS of *C. burnetii* and to determine its role in the bacterium's biology. During our investigation, we discovered that the S23p ORF is undergoing reductive evolution with variation in sequences immediately upstream of and within the ORF, in different strains of *C. burnetii*. We also discovered that, similar to other bacteria with IVSs in their 23S rRNA, the element is excised by RNase III. Moreover, consistent with previous findings, *Coxiella*'s IVS RNA was found to be labile following its excision from 23S rRNA (Burgin et al., 1990; Konkel et al., 1994). Together, these observations led us to hypothesize that *C. burnetii* IVS RNA might be physiologically neutral and its chief function is the physical fragmentation of 23S rRNA.

## Materials and methods

### Cultivation of *C. burnetii*

*C. burnetii* nine Mile phase II, clone 4 (strain RSA 439) were grown in acidified citrate cysteine medium 2 (ACCM-2; Omsland et al., 2011) using 0.2-μm-pore size filter-capped 500-ml Erlenmeyer flasks containing 80 ml of medium. *C. burnetii* was inoculated at a concentration of 1.6 × 10^4^ genome equivalents per ml (GE/ml) quantified by quantitative PCR (qPCR) with a primer set specific to the *C. burnetii rpoS* gene (Table S1; Coleman et al., 2004). Cultures were incubated at 37°C in a tri-gas incubator (2.5% O_2_, 5% CO_2_, 92.5% N_2_) with continuous shaking at 75 RPM (Omsland et al., 2011). Following 1 week of growth in the tri-gas incubator, flasks were capped and moved to room temperature for 21 d for generation of SCVs, as previously described (Sandoz et al., 2014).

### RNase III assay

The *C. burnetii* RNase III gene (*rnc*, CBU_1503; Primers: CbuRNaseIII_F and _R; Table S1) was cloned and the encoded, recombinant protein purified as previously described for the RNA helicase (*rhlE*) gene (Hicks et al., 2011). An RNase III assay substrate was prepared by T7 promoter-mediated *in vitro* transcription of a PCR product (from primers IVSflank+T7_F and IVSflank_R; Table S1) using a MAXIscript kit (Ambion), as instructed. The resulting RNA was electrophoresed in a 4% acrylamide (w/v)—8M urea gel, and the RNA was excised, eluted overnight at 37°C into a buffer [0.5 M ammonium acetate, 1 mM EDTA, and 0.2% sodium dodecyl sulfate (SDS) at pH 7] and then precipitated with 100% ethanol. RNase III assays were performed for 30 min at 37°C using 200 nM substrate RNA in RNase assay buffer (50 mM NaCl, 10 mM Tris, pH 7.9, 10 mM MgCl_2_, 1 mM DTT, 450 mM KCl) in the presence of recombinant *C. burnetii* RNase III (30 μM). Positive controls were done using *E. coli* RNase III (Ambion) as per manufacturer's instructions. Products of the RNase III assays were analyzed on 4% acrylamide (w/v)—8M urea gels.

### Identification of 5′ and 3′ ends of IVS

Termini of the IVS RNA were determined by 5′ and 3′ rapid amplification of cDNA ends (RACE) using a SMARTer RACE 5′/3′ kit, as instructed (Clontech). RNase III assay products were analyzed on 4% acrylamide (w/v)—8M urea gels from which IVS was purified, before resuspending in RNase-free water. Prior to 3′ RACE, IVS RNA was poly-adenylated at the 3′ end with *E. coli* Poly(A) polymerase, as instructed (NEB). cDNA was generated from IVS RNA before amplification by PCR using gene-specific primers (Table S1; IVS_RACE_GSP1, IVS_RACE_GSP2) and universal primers (provided with the kit). Further, a second nested PCR was performed on the 5′ RACE primary PCR product using IVS_RACE_NGSP1 (Table S1) and a universal short primer (provided with the kit) for increased resolution. PCR products were visualized on 2% agarose (w/v) gels, purified with a PCR clean-up and gel extraction kit as instructed (Clontech) and cloned into pRACE with an In-Fusion HD Cloning kit per manufacturer's instructions (Clontech). The plasmid content of individual clones was analyzed by automated Sanger sequencing at the Genomics Core Facility at the University of Montana.

### *In vitro* coupled transcription-translation (IVTT) of S23p

Translation of the IVS ORF encoding S23p was performed using an *E. coli* S30 Extract System for Linear Templates, as instructed by the manufacturer (Promega). Linear DNA templates were prepared by ligating a PCR-amplified tac promoter (*Ptac*) to the experimentally-determined 5′ end of the IVS of *C. burnetii* (RSA 439 and RSA 493) to generate S23p_439 and S23p_493, respectively (for Primers refer to Table S1). Additionally, an artificial template was engineered by including a ribosome binding site (RBS; AGGAGG) 7 bp upstream of the start codon of S23p before ligating the *Ptac* (for primers refer to Table S1). A FluroTech™ Green_Lys_ *in vitro* Translation Labeling system (Promega) was included in the reaction for fluorescent detection of translated proteins. Reactions were analyzed on 12.5% acrylamide (w/v) SDS-polyacrylamide gel electrophoresis (PAGE) gels and visualized using a FLA 3000G Fluorescent Imager (Fujifilm).

### Growth assays on *E. coli* expressing *Coxiella*'s S23p

This assay was performed essentially as previously described (Raghavan et al., 2008). Briefly, linear DNA templates (S23p+RBS, S23p_439, and S23p_493) used in IVTT reactions were cloned into pCR2.1-TOPO (Invitrogen), as per manufacturer's instructions, to generate pS23p+RBS, pS23p_439, and pS23p_493, respectively (see Table S2). *E. coli* (TOP10F′) was transformed with these plasmids to generate strains IRW201, IRW202, IRW203, respectively, or an empty vector (strain IRW204; Table S2) as instructed by the manufacturer (Invitrogen) and were grown overnight at 37°C in lysogeny broth (LB) broth containing 100 μg/ml ampicillin (LBamp). These cultures were used to inoculate fresh LBamp at a 1:10 (v/v) dilution, and the mixture was grown to mid-logarithmic phase (2 h) at 37°C. Isopropyl-beta-D-thiogalactopyranoside (IPTG) was added to 1 mM and growth was assayed spectrophotometrically at 600 nm at hourly intervals for 7 h.

### RNase protection assay (RPA)

The half-life of IVS RNA in *E. coli* was determined using a RPA. The IVS element and ~400 bp of flanking sequences were PCR amplified by standard protocol using IVSflank_F and _R primers (Table S1) and cloned into pCR2.1-TOPO per manufacturer's instructions (Invitrogen) to generate pIVS1. *E. coli* TOP10F' was transformed with pIVS1 to generate strain IRW205, which was cultured in LBamp for 16 h at 37°C and then used to inoculate LBamp at a 1:20 dilution. Following growth for 2 h at 37°C, IPTG was added to 1 mM and cultures were allowed to grow an additional 2 h. Rifampin was added to a final concentration of 160 μg/ml and 1 ml samples were taken at 5 min intervals for 35 min. RNA was purified from samples using TRI Reagent (Ambion) and processed using a RPA III kit (Ambion) [3 μg total RNA; 800 pg IVS probe (Table S3)], as previously described (Hicks et al., 2011). Biotinylated IVS RPA probes were prepared using a MEGAScript kit as instructed (Thermo Fisher), biotin-labeled UTP (Bio 16-UTP; Ambion), and corresponding IVS primers (Table S1; IVSprobe_F and IVSprobe_R+T7).

### Quantitative real-time PCR (qRT-PCR)

*In vivo* stability of IVS RNA in *C. burnetii* was determined by qRT-PCR. *C. burnetii* was cultured in ACCM-2, and DNA and RNA were isolated from the same flask at day 0, 1, 7, 10, 13, 16, 19, and 22 using a DNeasy Blood and Tissue kit (Qiagen) and Ribopure kit (Amibion), respectively, as per manufacturer's instructions. Resulting RNA was treated with TURBO DNase (Ambion), precipitated with 100% ethanol and quantified by spectrophotometry (260 nm). RNA (20 ng) from each growth time point was converted to cDNA using an iScript cDNA synthesis kit (Bio-Rad), as instructed by the manufacturer. Genome numbers were determined by qPCR as above. cDNA (diluted 100-fold) was used to perform qRT-PCR with corresponding primer sets (Table S1), as previously described (Raghavan et al., 2008). Amplified cDNA was normalized to respective genome numbers. In addition, total RNA was isolated from *C. burnetii* cultured in ACCM-2 (0–21 d) and processed as described above, except that 500 ng total RNA was converted to cDNA. qRT-PCR was performed on cDNA samples (diluted 10^4^-fold) using two unique primer sets specific to *C. burnetii*'s 16S, and IVS (Table S1). The qRT-PCR primers were designed using Accelrys gene software (Biovia).

### Northern blot analysis

Northern blots were carried out using a NorthernMax kit (Ambion) as per manufacturer's instructions. *C. burnetii* was cultured in ACCM-2, and total RNA was isolated at 1, 2, 3, 4, 5 d using TRI Reagent Solution (Ambion) following manufacturer's instructions. One hundred nanograms total RNA from each time point was electrophoresed through 1.5% agarose-formaldehyde gels and blotted onto positively-charged BrightStar-Plus nylon membrane (Ambion). Membranes were UV-cross-linked and hybridization were carried out using single-stranded rRNA probes specific to 16S, 23S F1, and 23S F2. rRNA probes were generated as previously described (Warrier et al., 2014). Membranes were developed with Chemiluminescent Nucleic Acid Detection Module Kit (Thermo Fisher) following manufacturer's protocol, and visualized using a LAS-3000 imaging system (Fujifilm). Densitometry was performed using ImageJ software (Schneider et al., 2012; Please see Tables S1 and S3, for primers and probe details).

## Results

### *In vitro* processing of *C. burnetii* IVS by RNase III

**Figure 1:**
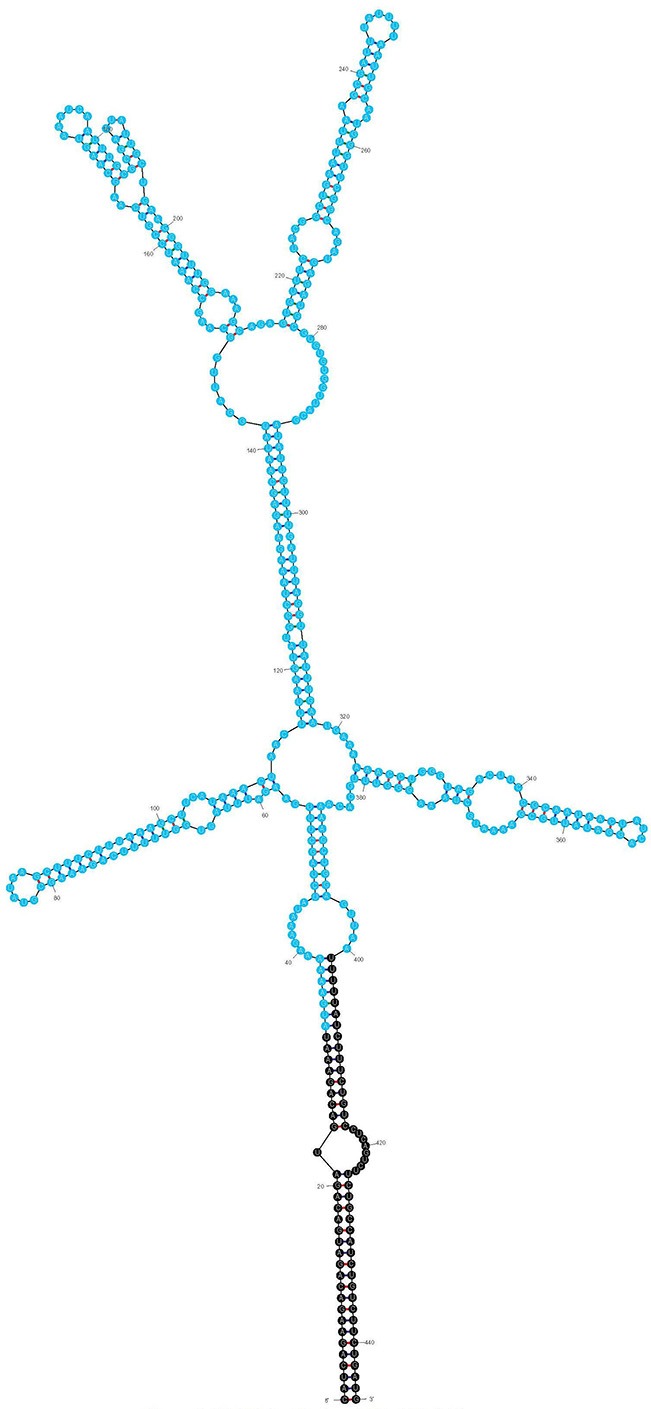
**Predicted secondary structure of the IVS using Mfold (Zuker, 2003; ΔG = −143.26 kCal/mol)**. An ORF (CBU_2096) encoding a potential S23p protein is shown in blue.

**Figure 2:**
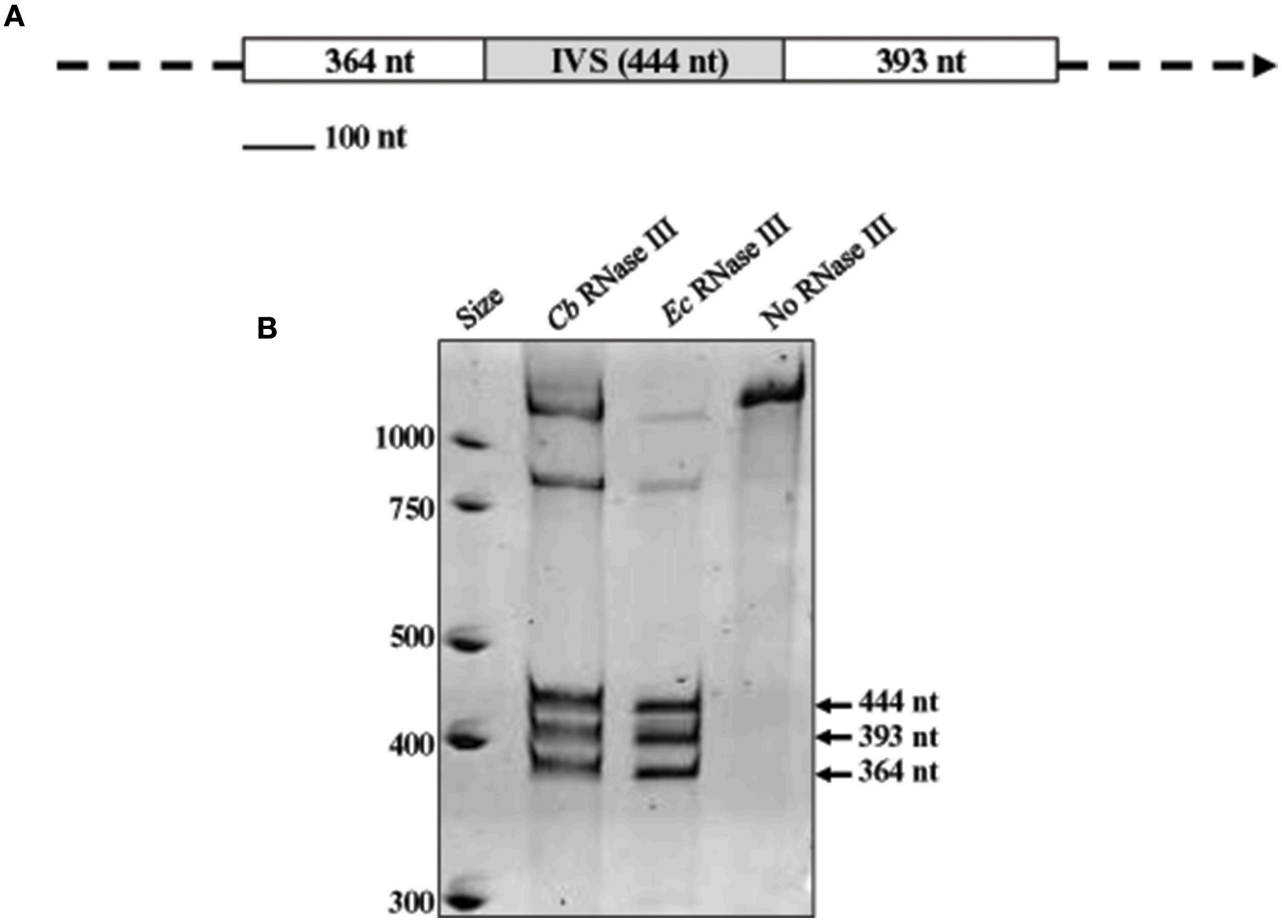
***In vitro* processing of the IVS by RNase III. (A)** Map of the RNA substrate used in RNase III assays, consisting of a segment of 23S rRNA with the IVS (gray box) and proximal flanking sequences (white boxes) with sizes (in nucleotides) shown. **(B)** Acridine orange-stained polyacrylamide gel [4% acrylamide (w/v)—8M urea] showing cleavage products of substrate RNA following treatment with recombinant *C. burnetii* (*Cb*) RNase III. Three discrete fragments of sizes corresponding to the substrate RNA (arrowed) were observed. Positive [*E. coli* (*Ec*) RNase III (Ambion)] and negative (no RNase III) control reactions are shown for comparison.

The IVS of *C. burnetii* was first described by Afseth et al. as a 444-nt element that is excised to yield a fragmented yet functional 23S rRNA (Afseth et al., 1995). The IVS element is located in helix 45 of *C. burnetii*'s precursor 23S rRNA. IVS RNA of *C. burnetii* (RSA 493) forms a stable stem-loop secondary structure (ΔG = −143.26 kCal/mol) due to the presence of complementary sequences at its termini (Figure 1). The terminal inverted repeats form a 28-bp stem with complete complementarity except for a single mismatch and a bulge. IVS elements of other bacteria are also associated with 23S rRNA genes and excised by RNase III (Burgin et al., 1990; Ralph and McClelland, 1993; Evguenieva-Hackenberg and Klug, 2000); an endoribonuclease that cleaves rRNA precursors during maturation (Dunn and Studier, 1973). To determine if RNase III is responsible for excision of IVS from the 23S rRNA precursor of *C. burnetii*, we performed an *in vitro* assay with T7-transcribed substrate RNA containing IVS and ~400 nt of flanking sequences (Figure 2A) and either recombinant *C. burnetii* RNase III or commercial *E. coli* RNase III. Since RNase III cleavage is nonspecific at low ionic strength (Dunn, 1976), we tested cleavage efficiencies at various ionic strengths (data not shown), and a final ionic concentration of 0.5 M (50 mM NaCl plus 450 mM KCl) was used in the reactions. When products of the RNase III assay were analyzed on polyacrylamide gels, three distinct RNA bands corresponding to IVS (444 nt) and its flanking sequences (393 and 364 nts) were observed (Figure 2B). These results indicate that *C. burnetii* RNase III is capable of cleaving IVS *in vitro* and most likely does so *in vivo*. Indistinguishable RNA fragments were observed in reactions containing *E. coli* RNase III but were absent in the negative control (Figure 2B).

### Characterization of the 5′ and 3′ ends of IVS

**Figure 3:**
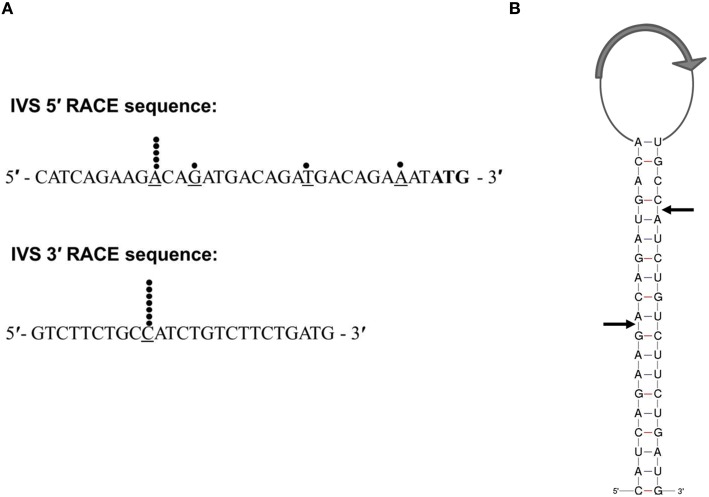
**Characterization of IVS termini by 5′- and 3′-RACE analysis. (A)** Sequences of the 5′ and 3′ ends of the IVS are shown. Black dots indicate the last base (underlined) in an individual sequence analysis of a cloned RACE PCR product. The predicted start codon of the S23p ORF is shown in bold font. **(B)** Schematic representation of RNase III processing sites on the stem of the IVS RNA of *C. burnetii* (RSA 493). Black arrows indicate processing sites as determined by 5′- and 3′-RACE analyses. The relative location of the S23 ORF (CBU_2096) is indicated by a large gray arrow in the loop.

Afseth et al. characterized the IVS length by identifying a sequence that was not a portion of the 23S rRNA by sequence homology (Afseth et al., 1995). To determine actual termini of the element, we performed 5′ and 3′ RACE analyses on IVS RNA isolated from the *Coxiella* RNase III *in vitro* assay. At least seven clones were isolated from each reaction and sequenced. The sequences of the 5′ and 3′ ends of IVS RNA are shown with black dots indicating experimentally-determined locations of the RNase III cleavage sites (Figure 3A). While the 3′ end of the IVS was uniform, the 5′ end showed a preferred cleavage site plus three minor, additional sites. When the sites of cleavage were mapped on the predicted secondary structure of IVS, they were found to be in the middle of the stem structure at about 9 bases from the 5′ end and about 15 bases from the 3′ end of the predicted IVS RNA sequence (Figure 3B). RACE analysis indicates the actual size of the *C. burnetii* IVS as 428 vs. 444 nt, as originally predicted (Afseth et al., 1995).

### Expression and evolution of the IVS ORF

**Figure 4:**
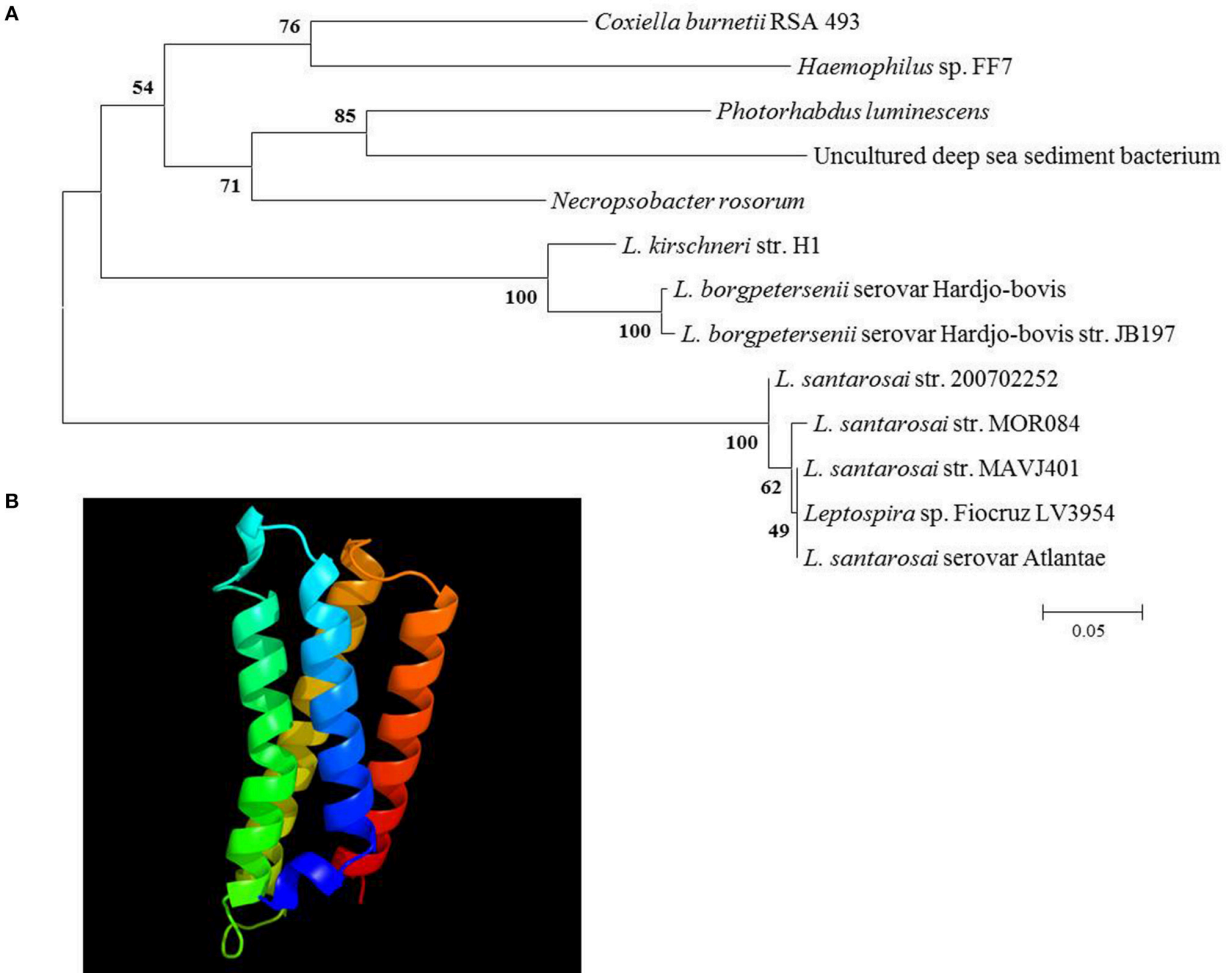
**Phylogenetic analysis and predicted secondary structure of the S23p. (A)** A neighbor-joining tree from predicted amino acid sequences of IVS ORF-encoded S23p superfamily proteins of the top 12 BLASTp hits to *C. burnetii*'s S23p. Bootstrap values (1000 replicates) are indicated at the nodes. Amino acid sequence identities ranged from 54 to 71% with *C. burnetii*'s S23p (*L*. = *Leptospira*). **(B)** Secondary structure of *C. burnetii*'s S23p as predicted by PHYRE2 (Kelley et al., 2015).

**Figure 5:**
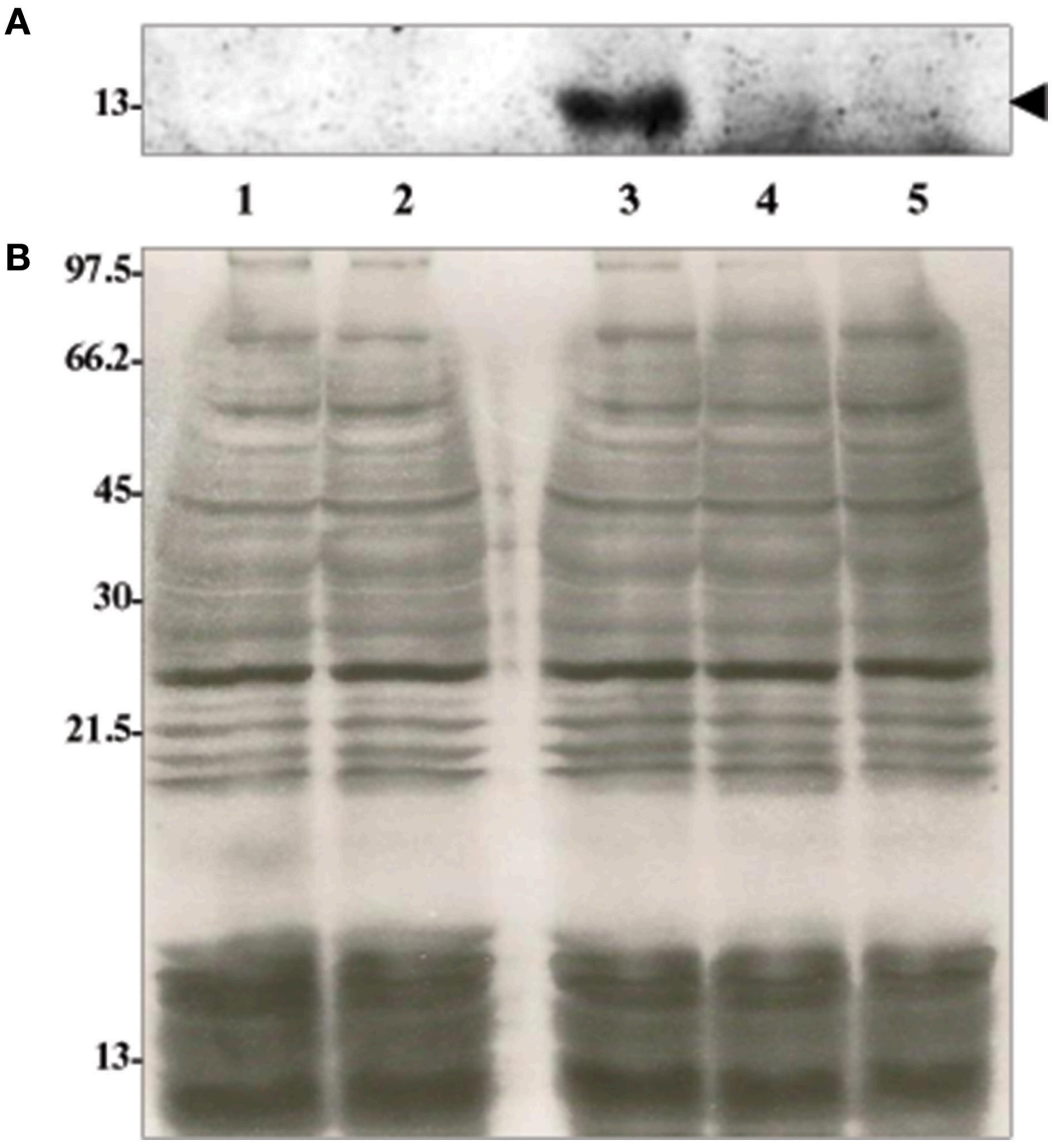
***In vitro* coupled transcription-translation (IVTT) of *C. burnetii*'s S23p. (A)** 12.5% acrylamide [wt/vol] SDS-PAGE gel visualized by fluorescent scanning. A prominent ~13-kDa protein band (arrow head) was identified in the IVTT reaction using S23p+RBS (Lane 3) but was absent in IVTT reactions using S23p_439 (Lane 4) and S23p_493 (Lane 5). **(B)** Corresponding silver-stained 12.5% acrylamide [wt/vol] SDS-PAGE showing products of IVTT reactions involving S23p+RBS (Lane 3), S23p_439 (Lane 4) and S23p_493 (Lane 5) as a template. A positive (from kit, Lane 1) and no-template control (Lane 2) are also shown. Molecular weight values from protein standards are indicated to the left in kDa.

**Figure 6:**
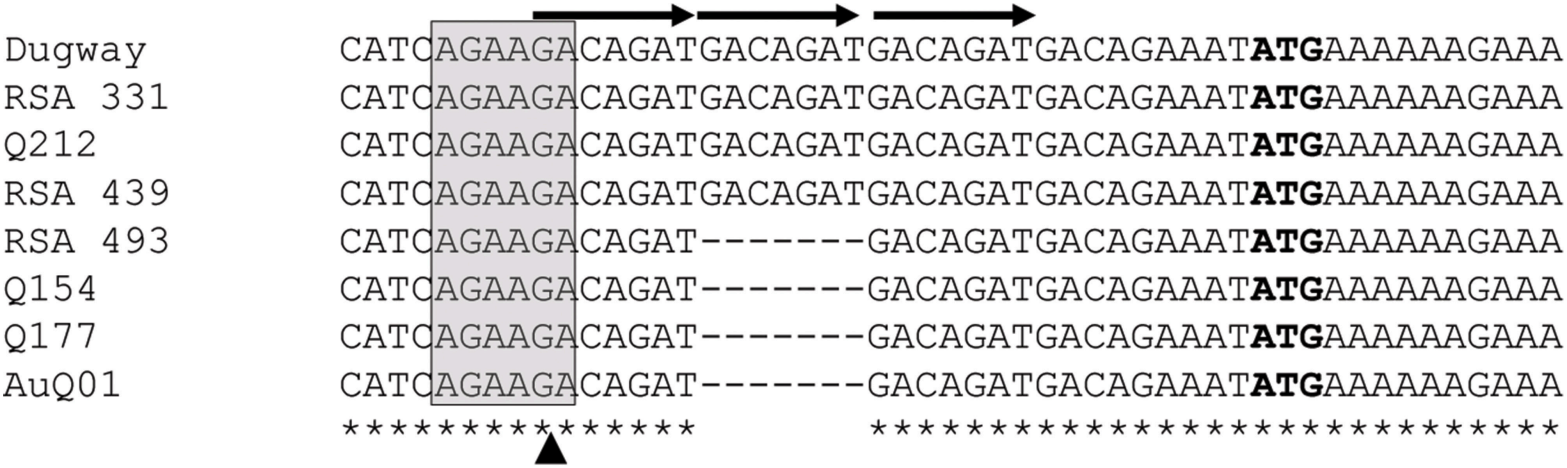
**Multiple sequence alignment of the 5′ region of *C. burnetii*'s IVS**. Corresponding sequences of selected *C. burnetii* strains (shown on the left; see Table S2) were aligned using ClustalX. Bases in the gray box represent a potential SD region. Arrows indicate direct heptameric repeats, while a dashed line shows deletion of one of the repeats. ATG (in bold) represents the start codon of the S23p ORF, as predicted by Afseth et al. (1995). An asterisk indicates perfect nucleotide sequence identity in the alignment. The arrowhead indicates the RNase III 5′ processing site.

The IVS of *C. burnetii* contains an ORF that encodes a predicted 14.2-kDa protein that belongs to the ribosomal S23 family of hypothetical proteins (S23p). While analyzing the nucleotide sequence of the S23p ORF, we found a true stop codon 63 nt downstream from the stop codon identified by Afseth et al. (1995). This observation was consistent with the NCBI database entry for the S23p ORF (CBU_2096; GeneID: 2820718). Phylogenetic analyses performed using the predicted amino acid sequence of *Coxiella*'s S23p, showed that the protein is most closely related to a homolog encoded in the IVS of *Haemophilus* sp. FF7, with 71% identity (Figure 4A). The predicted structure of *C. burnetii*'s S23p was determined using PHYRE2 (Kelley et al., 2015) and was found to consist of four α-helices (Figure 4B), similar to the S23p of *X. campestris* (Lin et al., 2006). As previously reported, a number of *C. burnetii* S23p homologs were also found in pathogenic *Leptospira* (Afseth et al., 1995). These results prompted us to determine whether the *C. burnetii* S23p protein could be expressed using an IVTT system. Linear DNA templates were prepared by engineering *Ptac* upstream of the experimentally-determined IVS sequences of *C. burnetii* RSA 439 and RSA 493 to produce S23p_439 and S23p_493, respectively. Additionally, an artificial template was constructed with a ribosome binding site (RBS; AGGAGG) upstream of the start codon in addition to *Ptac* to produce S23p+RBS. Following IVTT, protein products were resolved by SDS-PAGE and visualized by fluorescent imaging. An apparent ~13-kDa protein band was observed when S23p+RBS was used as template but was not detected in IVTT reactions using S23p_439 or S23p_493 (Figure 5A). A corresponding silver-stained SDS-PAGE (Figure 5B) shows equal loading of IVTT reactions in the lanes. These results suggest that the IVS ORF has the potential to synthesize a protein, but only in the presence of a strong RBS. In fact, the putative AG-rich SD region (AGAAGA; Figure 6) is removed from the IVS during excision by RNase III (see Figure 3A) implying that this protein is probably not expressed *in vivo*.

*In silico* analysis of the IVS regions of ~80 *C. burnetii* strains showed that some possess deletions in the sequence immediately upstream of the S23p ORF (Frangoulidis and Walter, unpublished data). As a rule, the 5′ region of the IVS consisted of a potential SD sequence (AGAAGA) followed by three, tandem heptameric repeats (GACAGAT) upstream of the start codon of the S23p ORF (Figure 6). An additional heptameric repeat with a degenerate nucleotide at its 3′ end (GACAGAA) occurred prior to the predicted ATG of the S23p ORF (Figure 6). An unequal number of heptameric repeats was observed upon analysis of different strains of *C. burnetii*. In Dugway, RSA 331, Q212, and RSA 439 strains, three sets of the heptameric repeat units were found, whereas in strains RSA 493, Q154, Q177, and AuQ01 only two sets of the repeat were present (Figure 6). Interestingly, two non-synonymous nucleotide substitutions were also observed within the S23p ORF in *C. burnetii* strains Dugway and Q212 at positions 203 and 291, respectively. In addition, the *C. burnetii* strain AuQ01 was found to have a large, 176-bp deletion in its S23p ORF (data not shown). Taken as a whole, the non-synonymous substitutions and large deletion suggest that the protein-coding segment of the IVS is undergoing negative selection and is expendable.

**Figure 7:**
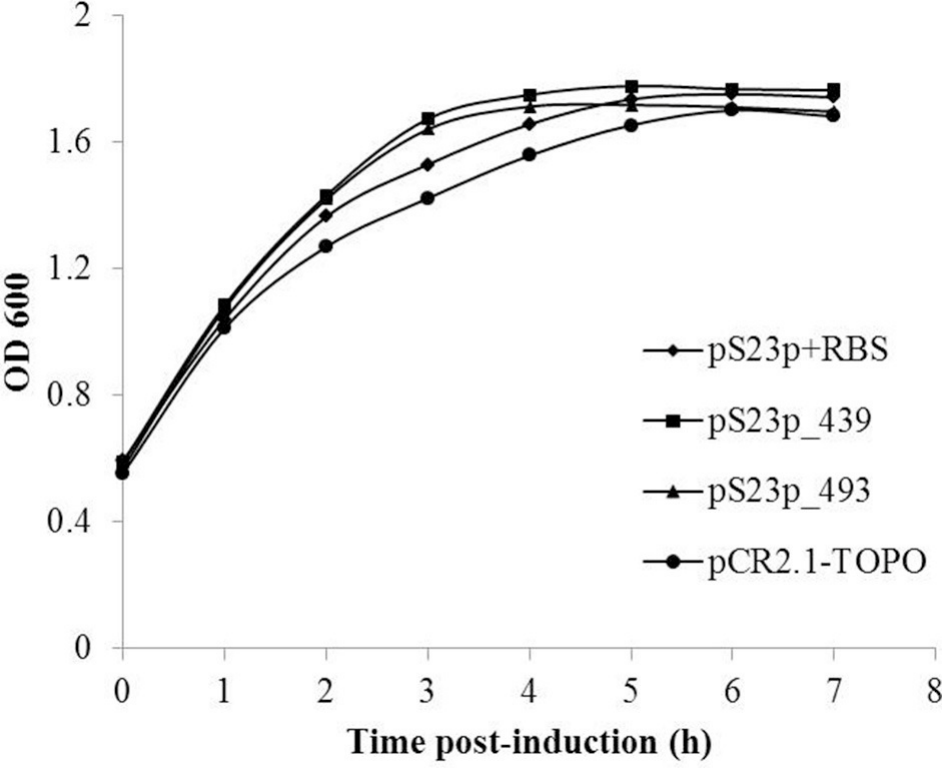
**Effect of *C. burnetii*'s S23p on *E. coli's* growth**. *E. coli* strains IRW201 (diamond, pS23p+RBS), IRW202 (square, pS23p_439), IRW203 (triangle, pS23p_493), IRW204 (circle, pCR2.1-TOPO vector) were induced with IPTG (1 mM) and assayed spectrophotometrically at 600 nm for growth at 37°C. A representative of two indistinguishable assays is shown.

In order to determine if expression of S23p affects the growth of *E. coli*, as previously observed with two Group I introns encoded in *C. burnetii*'s 23S rRNA gene (Raghavan et al., 2008), we cloned the linear templates used in the IVTT assay into pCR2.1-TOPO to produce plasmids pS23p+RBS, pS23p_439, and pS23p_493, respectively. Respective *E. coli* strains harboring these plasmids (i.e., IRW201, IRW202, IRW203) or the vector alone (IRW204) (see Table S2) were induced with IPTG and their growth rates monitored spectrophotometrically for 7 h. As shown in Figure 7, growth rates of *E. coli* expressing S23p (e.g., strain IRW201) were not significantly different from *E. coli* containing the pCR2.1-TOPO cloning vector alone (strain IRW204). This observation is consistent with a previous report showing that *E. coli* expressing the IVS of *S. typhimurium* did not display any growth defects (Gregory et al., 1996).

### *In vivo* stability of IVS RNA

**Figure 8:**
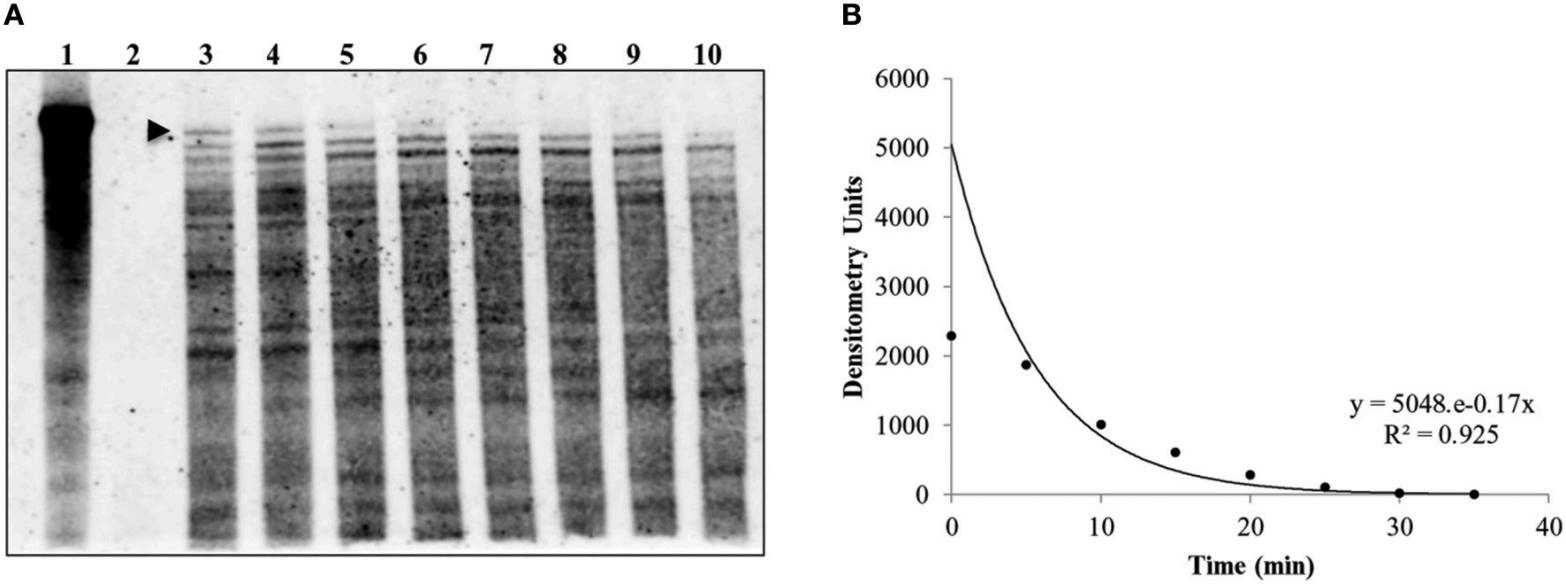
**Half-life of *C. burnetii*'s IVS RNA in *E. coli* (IRW205)**. A representative RPA used to determine the *in vivo* IVS half-life is shown. **(A)** RPA blot with IVS band (480 bases) indicated by an arrowhead (3 μg RNA per lane and 800 pg of IVS probe were used). Lanes 1 and 2 show controls with biotin-labeled IVS probe in the absence or presence of RNase A/T1, respectively; Lanes 3–10 show RNA isolated at 5-min intervals from 0 to 35 min following rifampin treatment. **(B)** Densitometric analysis of IVS bands in blot lanes 3–10 vs. time of collection following rifampin treatment. The equation for the exponential curve and the *R*^2^ value are inset and yield an IVS half-life of 3.9 min.

**Figure 9:**
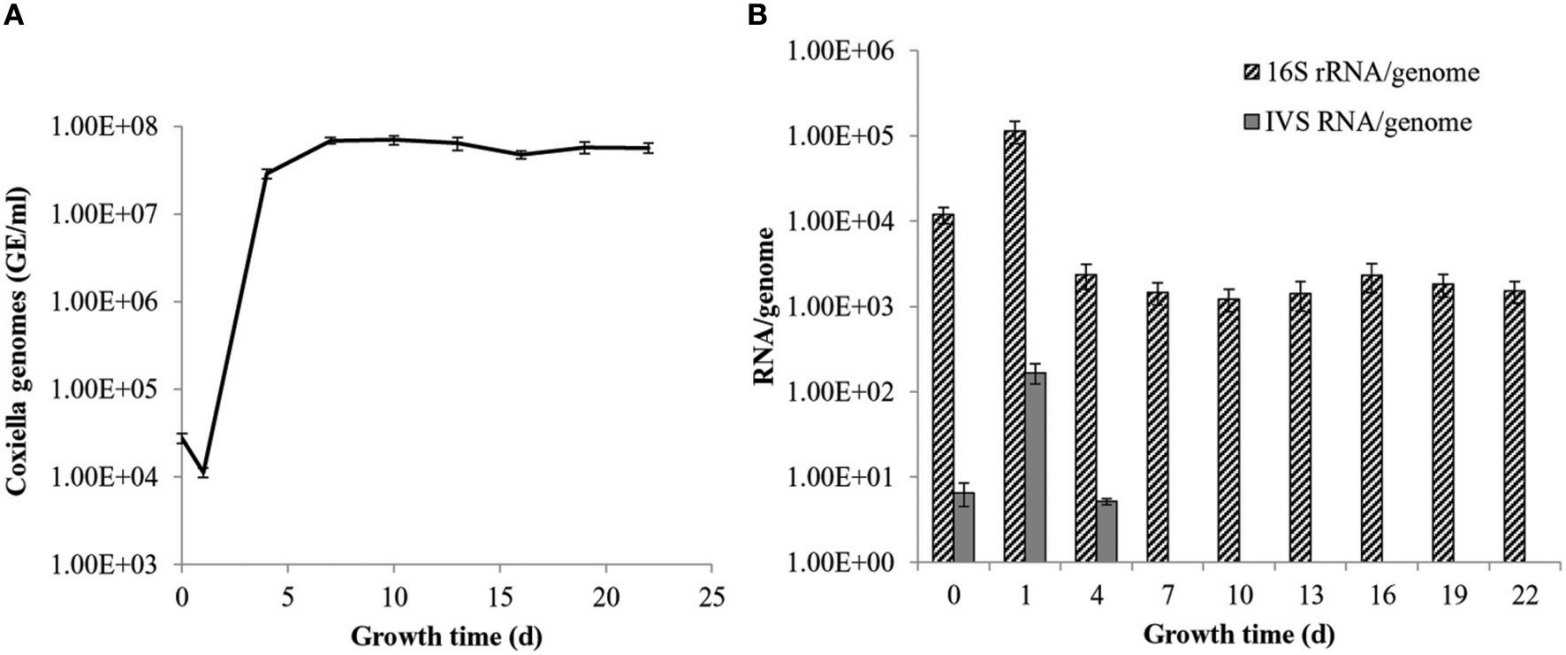
**Levels of IVS RNA in *C. burnetii* over time of growth. (A)** q-PCR data showing *C. burnetii* genome equivalents per ml (GE/ml) during growth in ACCM-2. **(B)** qRT-PCR data showing numbers of 16S rRNAs (striped bars) and IVS RNAs (gray bars) on a per genome basis. Data represent the means of six independent experiments ± S.D., except IVS data at 0 d, which represent the mean of three determinations ± S.D.

Previous work by our laboratory showed that *Coxiella*'s Group I introns, Cbu_L1917, and Cbu_L1951, were spliced from the precursor 23S rRNA and demonstrated remarkable stability (~11 min) in an *E. coli* background, suggesting a possible adaptive role in *Coxiella*'s biology (Hicks et al., 2011). Since the IVS is uniformly present and highly conserved among strains of *C. burnetii*, an adaptive role might also be possible for IVS RNA. To determine the intrinsic stability of the IVS RNA, we performed an *in vivo* stability assay in an *E. coli* model. *E. coli* IRW205 was cultured, expression induced by IPTG, and cells sampled after transcription was stopped with rifampicin. RPAs performed on RNAs isolated from these samples showed the half-life of IVS RNA to be ~4 min (Figure 8). Considering that the global, average half-life of mRNA in *E. coli* is ~5.7 min (Bernstein et al., 2002), these results suggest that IVS RNA does not display extraordinary intrinsic stability like *Coxiella*'s introns. However, since the RNase activities of *E. coli* may differ from *C. burnetii*, we also quantified levels of IVS RNA in *C. burnetii*. *C. burnetii* was cultured in axenic medium for 22 d and IVS RNA levels were measured by performing qRT-PCR on total RNA purified from the bacterium at 72-h intervals, using primers specific to IVS (Primers: IVS_qPCR_F and _R; Table S1). qPCR was also performed on genomic DNA isolated at the same time points (Figure 9A) to normalize the amount of IVS RNA on a per-genome basis. Results showed that IVS RNA was highest at 1 d (Figure 9B), i.e., the beginning of exponential phase (Omsland et al., 2011). Since rRNAs are highly transcribed during this growth phase (e.g., see the commensurate increase in 16S rRNA in Figure 9B) and mature 23S rRNA is formed following excision of IVS, the increased level correlates with increased synthesis of 23S rRNA. As the bacterium approached stationary phase (~4 d; Omsland et al., 2011), levels of IVS significantly declined and subsequently became insignificant (Figure 9B). Therefore, these results suggest that soon after excision from the 23S rRNA, IVS RNA is degraded in *Coxiella*, as observed in *E. coli* (Figure 8).

### Differential levels of 23S rRNA fragments following IVS excision

**Figure 10:**
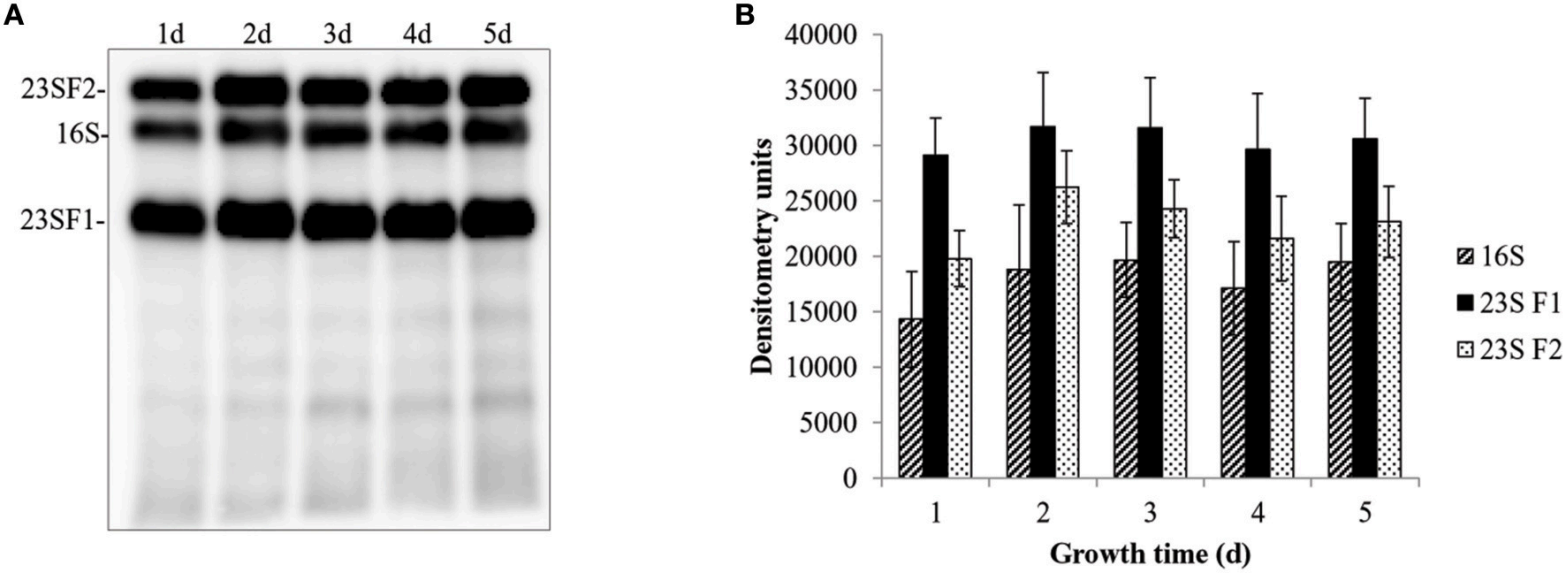
**Differential levels of *C. burnetii* rRNA species during growth**. Levels of 16S rRNA and two fragments of 23S rRNA (23S F1 and 23S F2) were determined by Northern blot analyses. **(A)** Representative example of a Northern blot showing 23S F2, 16S rRNA, and 23S F1 performed on total RNA isolated from *C. burnetii* cultured for 1–5 d in ACCM-2 (In each case, 100 ng of total RNA and 0.05 mM probes were used). **(B)** Densitometric analysis of 16S rRNA (striped bars), 23S F1 (black bars), and 23S F2 (white, speckled bars). Values represent the means ± S.D. of the result of 3 independent Northern blot analyses from 2 different RNA preparations.

The 23S rRNA of eubacteria is highly conserved, however, fragmentation caused by IVS elements does not appear to affect ribosome function (Evguenieva-Hackenberg and Klug, 2000). In fact, IVS elements occur within the 23S rRNA genes of several bacteria, including *Leptospira* (Ralph and McClelland, 1993), *Salmonella* (Burgin et al., 1990), and *Yersinia* (Skurnik and Toivanen, 1991). Although fragmentation does not affect the viability of these bacteria, it has been shown to affect the rate of 23S rRNA degradation in *Salmonella* as cells reach stationary phase (Hsu et al., 1994). Indeed, there appears to be a direct correlation between the degree of IVS-mediated fragmentation and the rate of 23S rRNA degradation, conferring a selective advantage to the bacterium. A similar adaptation could conceivably be adaptive to *C. burnetii* as it transitions to the small, spore-like, SCV. To investigate this possibility and to help elucidate the biological function of IVS, we performed Northern blot analyses to compare quantities of 16S rRNA to the two fragments of 23S rRNA from *C. burnetii* cultured in ACCM-2. As a result of RNase III-mediated IVS excision, the 23S rRNA of *C. burnetii* is cleaved into fragment 1 (F1, ~1.2 kb) and fragment 2 (F2, ~1.7 kb). Since *C. burnetii*'s structural rRNAs are all encoded by a single-copy operon, their concentrations would be expected to initially be at parity following transcription. However, we found that quantities of 23S F1 were consistently and significantly higher (*P* < 0.043) than 16S rRNA while the levels of 23S F2 were not significantly different from those of the 16S rRNA (Figure 10).

## Discussion

In the current study, we characterized the IVS of *C. burnetii*. IVSs of eubacteria usually occur within helix 9, 25, or 45 of the 23S rRNA (Ralph and McClelland, 1993; Evguenieva-Hackenberg and Klug, 2000; Pabbaraju et al., 2000). Conservation of the location of IVSs in 23S rRNA indicates that the continuity of 23S rRNA in these regions is not essential. IVSs are transcribed as part of the precursor 23S rRNA, excised during rRNA maturation and subsequently degraded. Unlike introns, the pieces of 23S rRNA remain fragmented after IVS excision, but still maintain the functional integrity of the ribosome by secondary and tertiary structures. Although 23S rRNAs are highly conserved among eubacteria, the occurrence of IVSs within 23S rRNAs is sporadic (Burgin et al., 1990; Ralph and McClelland, 1993; Mattatall and Sanderson, 1996). The scattered distribution of IVSs among bacteria suggests the possibility of horizontal transfer as a source for these elements. Horizontal gene transfer is known to be an important process in evolution with well-established roles such as acquisition of antibiotic-resistance genes (Krawiec and Riley, 1990) and movement of Group I and II introns between species (Dujon, 1989). In fact, the phylogeny of IVSs in *Leptospira* show evidence of natural horizontal transfer between species (Ralph and McClelland, 1994). Recently, Duron et al. suggested that all known strains of *C. burnetii* evolved from a *Coxiella*-like endosymbiont hosted by soft ticks of the *Ornithodoros* and *Argas* genera (Duron et al., 2015). In contrast, studies by Smith et al. and Gottlieb et al. indicated that *C. burnetii* and *Coxiella*-like endosymbionts of ticks originated from a common (possibly tick-associated) ancestor (Gottlieb et al., 2015; Smith et al., 2015). Regardless of evolutionary route, it is possible that a *C. burnetii* ancestor may have acquired virulence genes and metabolic genes via horizontal gene transfer (Duron et al., 2015; Gottlieb et al., 2015). Keeping this in mind, it is conceivable that *C. burnetii* initially acquired the IVS through horizontal transfer. Indeed, the GC content of *C. burnetii*'s IVS (~36%) relative to the remainder of the genome (~42.5%) underscores this possibility. Interestingly, none of the *Coxiella*-like endosymbionts were found to possess an IVS by BLAST searches (data not shown), suggesting that the element was acquired after *C. burnetii*'s divergence from endosymbionts. In contrast, when the occurrence of IVS among different strains of *C. burnetii* was analyzed in this study, it was found to be present in all available genomes with 99% sequence identity (data not shown). This observation suggests that *C. burnetii* strains acquired the element by vertical transfer from a common ancestor and it has been maintained by playing an adaptive or neutral role in the context of the intracellular niche.

Previous studies have shown that IVS is excised by RNase III, an endoribonuclease that cleaves rRNA precursors during maturation (Burgin et al., 1990; Ralph and McClelland, 1993; Evguenieva-Hackenberg and Klug, 2000). Using an *in vitro* RNase III assay, we showed that *C. burnetii* RNase III is capable of cleaving IVS *in vitro* and most likely *in vivo* (Figure 2). Additionally, we determined the actual termini of the IVS element of *C. burnetii* by 5′ and 3′ RACE analysis, which were mapped on the predicted secondary structure (Figure 3B). The true ends of IVS were found to be in the middle of the stem structure formed by inverted repeats, indicating the actual size of the *C. burnetii* IVS as 428 vs. 444 nt, as originally predicted (Afseth et al., 1995). Following cleavage, the remaining stem portion of the IVS could either be retained in the mature 23S rRNA that holds the rRNA pieces together, as observed in *Leptospira* (Ralph and McClelland, 1993; Konkel et al., 1994), or it could be trimmed by exonucleases. One example of such a ribonuclease is RNase T, which plays a role in 3′ maturation of *E. coli*'s 23S rRNA and is also present in *C. burnetii* (Deutscher, 2009). However, an exoribonuclease involved in maturation of 5′ ends has not yet been identified (Deutscher, 2009).

Another interesting feature of *C. burnetii* IVS is the presence of an ORF that potentially encodes for a 14.2-kDa protein that belongs to the ribosomal S23p family. A similar ORF within the IVS has previously been found in a few bacteria, including *Leptospira, Xanthomonas*, and *Brucella* (Ralph and McClelland, 1993; Lin et al., 2006). Since the ORF is oriented such that the sense strand is present in the 23S rRNA primary transcript, it would be one of the most abundantly transcribed RNAs in the cell with the potential to be translated. Interestingly, we found that IVS ORF of *C. burnetii* could express a 13-kDa protein *in vitro*, but only in the presence of a strong *E. coli* tac promoter and RBS (Figure 5A). Since the AG-rich SD region of *C. burnetii* IVS is removed during RNase III excision, S23p is probably not expressed *in vivo*. In agreement with our data, S23p has not been identified, to date, in proteomic analyses of *C. burnetii* (Coleman et al., 2007; Skultety et al., 2011). When the sequence of the IVS ORF of *C. burnetii* was compared among species, considerable sequence variation was observed (Figure 6). The sequence immediately upstream of the S23p ORF's start codon and the S23p ORF itself showed deletions and non-synonymous substitutions suggesting that the protein coding region is undergoing reductive evolution. In spite of the variable number of heptameric repeats observed in the region upstream of IVS (Figure 6), an RNase III cleavage site would presumably be available since it occurs at the beginning of the first repeat and there are several direct repeat elements. These results lead us to hypothesize that the RNA component of the IVS is more biologically relevant to *C. burnetii* than S23p.

Although IVS was identified over 25 years ago as the basis for enhanced fragmentation of 23S rRNA in *Salmonella typhimurium* and *S. arizonae* (Burgin et al., 1990), the functionality and origin of these elements remain unclear. One among many hypotheses is that IVSs could be remnants of a transposition event (Burgin et al., 1990). This would explain the presence of inverted repeats, since they are commonly found as a result of insertion and subsequent excision of a transposable element (Kleckner, 1981). Another theory is that IVS was a mobile genetic element where the ORF coded for a component required for mobility and which also conveyed a selective advantage to the host (Ralph and McClelland, 1993). Molecular evidence suggests that *C. burnetii* recently shifted from a free-living to an obligate intracellular lifestyle and the genome is undergoing reductive evolution (Seshadri et al., 2003; Minnick and Raghavan, 2011). Therefore, it is conceivable that the IVS might have had such a history in *C. burnetii*.

With respect to functionality, we, among others, have found that the IVS has no detectable physiological consequence to the host, apart from fragmentation of 23S rRNA. For example, *E. coli* expressing a 23S rRNA that contains *S. typhimurium*'s IVS causes fragmented 23S rRNA, but maintains a wild-type growth rate indicating a phenotypically silent effect (Gregory et al., 1996). Similarly, *E. coli* expressing *C. burnetii*'s IVS or S23p did not show any growth defects. We also observed that IVS RNA in *C. burnetii* was only detectable during the growth phase of the bacterium, which corresponds to increased synthesis and maturation of 23S rRNA (Figure 9B). Similar results were observed when the intrinsic stability of IVS RNA was determined in an *E. coli* model (Figure 8). These observations are consistent with previous reports for IVS elements of other bacteria, where IVS RNAs were not detectable by Northern blot analysis during growth, indicating a reduced stability following their excision from a 23S rRNA precursor (Burgin et al., 1990; Konkel et al., 1994). Taken together, our results suggest that soon after excision from 23S rRNA, IVS RNA is degraded in *Coxiella*. However, when quantities of the two fragments of 23S rRNA, following IVS excision, were compared to 16S rRNA, we found that 23S F1 was significantly greater than levels of 16S rRNA while 23S F2 was not (Figure 10). The differential stability of F1 and F2 was surprising, since studies in *Salmonella* have shown that both fragments of its 23S rRNA are equally prone to degradation near stationary phase (Hsu et al., 1994). Since rRNA transcription, maturation and ribosome assembly are coupled in bacteria, it is conceivable that fragmentation of 23S rRNA might occur in the context of the ribosome. In such a case, following excision of IVS, F1 might be incorporated into the ribosome and protected from RNase degradation. In contrast, F2 might be more labile since it is relatively larger in size than F1 (~1.7 vs. ~1.2 kb, respectively) and has two introns that must also be spliced out during maturation. However, the exact reason and consequence for increased stability of 23S F1 is yet to be determined. These results suggest that following transcription and during maturation, each rRNA fragment experiences a unique micro-environment that determines its ultimate fate.

## Author contributions

Conceived and designed the experiments: IW, RR, MW, MM. Performed the experiments: IW, LH, MW. Analyzed the data: IW, MW, DF, RR, LH, MM. Wrote the paper: IW, MM.

### Conflict of interest statement

The authors declare that the research was conducted in the absence of any commercial or financial relationships that could be construed as a potential conflict of interest.
