# Supplementary material for: The Intervening Sequence of Coxiella burnetii: Characterization and Evolution
Source: Front Cell Infect Microbiol. 2016 Aug 19;6:83. doi: 10.3389/fcimb.2016.00083 (PMC4990558; doi:10.3389/fcimb.2016.00083)
Supplement: Supplementary file 1 [file Table1.pdf]

Table S1. Primers used in the study and their respective targets.

Target	Designation	Sequence
rnc	CbuRNaseIII_F	TTTTGGATCCAACCATCTTAACAAGTTA
	CbuRNaseIII_R	TTTTCCCGGGTCATTGGTCCCGCTCCGT
IVS	IVSflank+T7_F	TAATACGACTCACTATAGGGATAGCTGGTTCTCCTCG
	IVSflank_F	GATAGCTGGTTCTCCTCG
	IVSflank_R	CTTTTCCTGGAAGCGTGG
	IVS_RACE_GSP1	GATTACGCCAAGCTTACCACACACGCATCTCATCTGCCGAAC
	IVS_RACE_GSP2	GATTACGCCAAGCTTTGGATTGGCAAGCCAAATCCGTCAAGCAAG
	IVS_RACE_NGSP1	GATTACGCCAAGCTTGAAACACTCTGCTTTCCAAACCCTTCAGC
	IVSprobe_F	TTTTTTTTTTTTTTGTGTACCGAAGCTGCGG
	IVSprobe_R+T7	TAATACGACTCACTATAGG TTTTTTTTTTTTTTACAGAACGCTCCTCTACC
	IVS_qPCR_F	ATTGCAATGGGTTCGGCAGATGAG
	IVS_qPCR_R	ACAGATGGCAGAAGACTGAGGACA
S23p ORF (Cbu_2096)	S23p+RBS+BamHI_F	GGATCCTTAGGAGGTTTTTTTATGAAAAAAGAAATATCTAG
	S23p_439+BamHI_F	GGATCCTTACAGATGACAGATGACAGATGACAGAAATATGA
	S23p_493+BamHI_F	GGATCCTTACAGATGACAGATGACAGAAATATGA
	S23p_R	GGCAGAAGACTGAGGACAGAAAGATAAAAATTAAC
Ptac	*Ptac*_F	GAGCTGTTGACAATTAATCATCGGCTC

promoter	*Ptac*+BamHI_R	AAGGATCCAATGTTTCCTGTGTGAAATTG
rpoS	QrpoS_F	CGCGTTCGTCAAATCCAAATA (Coleman et al., 2004)
	QrpoS_R	GACGCCTTCCATTTCCAAAA (Coleman et al., 2004)
CBU_16S	16SqPCR_F	TTCGGGAACCGAGTGACAGGTG
	16SqPCR_R	TCGCTGGCAACTAAGGACGAGG
	16Sprobe_F	GTAATGCGTAGGAATCTACC
	16Sprobe_R	TAATACGACTCACTATAGGGATCCGACTTAAATATCCACC
CBU_23S	23SF1probe_F	CTGGAAAGTTCAGCCATAGC
	23SF1probe_R	TAATACGACTCACTATAGGGGCCTTTCACTCCTATCCACAG
	23SF2probe_F	AAAACCCACACGCCGAAAG
	23SF2probe_R	TAATACGACTCACTATAGGGAGCCACCTGGTATCTGCAAC

*The T7 promoter sequence is underlined

Table S2. Plasmids, genomes and strains used in the study.

**Plasmids, genomes, strains**	**Relevant characteristics**	**Source or reference**
**Plasmids**		
pCR2.1-TOPO	TA cloning vector	Invitrogen
pRACE	Cloning vector for RACE analysis	Clontech
pS23p+RBS	pCR2.1-TOPO containing S23p ORF with *Ptac* promoter and ribosome binding site upstream	This study
pS23p_439	pCR2.1-TOPO containing S23p ORF of *C. burnetii* RSA 439 with *Ptac* promoter	This study

pS23p_493	pCR2.1-TOPO containing S23p ORF of *C. burnetii* RSA 493 with *Ptac* promoter	This study
pIVS1	pCR2.1-TOPO containing IVS element and ~400 bp flanking sequences	This study

***Coxiella* genomes**		
RSA 493	Nine Mile phase I; tick isolate, 1935	(Seshadri et al., 2003)
RSA 439	Nine Mile phase II, clone 4; tick isolate, 1935	(Seshadri et al., 2003)
Dugway	Attenuated rodent isolate, (5J108-111), 1958	(Beare et al., 2009)
RSA 331	Human blood isolate (Henzerling), 1945	NC_010117
Q212	Human heart valve isolate (G), 1981	(Beare et al., 2009)
Q154	Human heart valve isolate (K), 1976	(Beare et al., 2009)
Q177	MSU goat isolate (Priscilla), 1980	NZ_AAUP02000006
AuQ01	Human serum isolate, acute Q fever, 2014	(Walter et al., 2014)

E. coli strains		
TOP10F'	TOP10F'	Invitrogen
IRW201	TOP10F' carrying pS23p+RBS	This study
IRW202	TOP10F' carrying pS23p_439	This study
IRW203	TOP10F' carrying pS23p_493	This study
IRW204	TOP10F' carrying pCR2.1-TOPO	This study
IRW205	TOP10F' carrying pIVS1	This study

Table S3. Probes used in Northern blots and RPAs.

**Probes**	**Sequence 5' to 3'**
IVS	TTTTTTTTTTTTTTGTGTACCGAAGCTGCGGCATCAGAAGACAGATGACAG ATGACAGAAATATGAAAAAGAAATATCTAGCTTTGAAGATTTAGTGGTT TTTCAGAAAGCGTATAAGCTTTCTTTAAAGCTTCATAAACTAACTTTAAGT ATGCCTAAAGAAGAGCAATATGGATTGGCAAGCCAAATCCGTCAAGCAAG TAAATCAATTTGCGCGAATATTGCTGAAGGGTTTGGAAAGCAGAGTGTTTC

	TACGGCTGAATTTAAGCGATTTATTTTAATTGCAATGGGTTCGGCAGATGA<br>GATGCGTGTGTGGTTACGATATTGTTTTGATTTAGGTTATTTGAATGAAAA<br>AACCTGGGTAGACTTCCGCAATAACTATCAGGATATTGCGAAAATGCTAA<br>AGGGTTTGCACAAAAGCTGGAGTTAATTTTTATCTTTCTGTCCTCAGTCTTC<br>TGCCATCTGTCTTCTGATGGGTAGAGGAGCGTTCTGTTTTTTTTTTTTTT
16S	GTAATGCGTAGGAATCTACCTTGTAGTGGGGGATAACCTGGGGAAACTCG<br>GGCTAATACCGCATAATCTCTTTGGAGCAAAGCGGGGGATCTTCGGACCTC<br>GTGCTATAAGATGAGCCTACGTCGGATTAGCTTGTTGGTGGGGTAATGGCC<br>TACCAAGGCGACGATCCGTAGCTGGTCTGAGAGGACGATCAGCCACACTG<br>GGACTGAGACACGGCCCAGACTCCTACGGGAGGCAGCAGTGGGGAATATT<br>GGACAATGGGGGAAACCCTGATCCAGCAATGCCGCGTGTGTGAAGAAGGC<br>CTTCGGGTTGTAAAGCACTTTCGGTGGGGAAGAAATTCTCAAGGGTAATAT<br>CCTTGGGCGTTGACGTTACCCACAGAAGAAGCACTGGCTAACTCTGTGCCA<br>GCAGCCGCGGTAATACAGAGAGTGCAAGCGTTAATCGGAATCACTGGGCG<br>TAAAGCGCGCGTAGGTGGATATTTAAGTCGGAT
23S F1	CTGGAAAGTTCAGCCATAGCGGGTGATAGCCCCGTACGCGAAAGAGTAAA<br>TAATGTGGGTAACGATGAGTAGGTCGGGACACGTGGTATCTTGACTGAAC<br>ATGGGGGGACCATCCTCCAAGGCTAAATACTCCTTACTGACCGATAGCGA<br>ACCAGTACCGTGAGGGAAAGGTGAAAAGAACCCCGGCGAGGGGAGTGAA<br>ATAGAACCTGAAACCGTATGCGTACAAGCAGTAGGAGCATTTCTTCGGAA<br>ATGTGACTGCGTACCTTTTGTATAATGGGTCAGCGACTTACTTGTTGTAGC<br>GAGCTTAACCGTCTAGGGGAGGCGTAGGGAAACCGAGTCCGAAATGGGCG<br>TTTAGTTGCAACGAGTAGACCCGAAACCGAGCGATCTATCTATGGCCAGG<br>GTGAAGGTCAGGTAACACTGACTGGAGGCCCGAACCCACTAATGTTGAAA<br>AATTAGGGGATGAGCTGTGGATAGGAGTGAAAGGC
23S F2	AAAACCCACACGCCGAAAGTCTAAGGTTTCCTGCGCAACGTTAATCGACG<br>CAGGGTGAGTCGGCCCCTAAGGCGAGGCAGAAATGCGTAGTCGATGGGAA<br>ACGGGTTAATATTCCCGTACTTTATAATACTGCGATGGGAGGACGGAGAA<br>GGCTAGGTCAGCCACCCGATGGTTGTGGTGGTTTAAGTGTGTAGGAAGGG<br>TTCTTTGGCAAATCCGGGAACTCAATTCCGAGACATGATGACGAAGTACG<br>AACTTGTTCGTGCAAAGTGATTGATGCCACGCTTCCAGGAAAAGTCCCTAA

GCTTCAGGTATTGTAAAACCGTACTATAAACCGACACAGGTGGACAGGTA
GAGAATACCAAGGCGCTTGAGAGAACTTGGGTGAAGGAACTAGGCAAAA
TGGCACCGTAACTTCGGGAGAAGGTGCGCCCTTGGTAAGTGAAGGTCCTT
GCGACTGGAGCTGAAAAGGGTTGCAGATACCAGGTGGCT

REFERENCES:

Beare, P.A., Unsworth, N., Andoh, M., Voth, D.E., Omsland, A., Gilk, S.D., et al. (2009). Comparative genomics reveal extensive transposon-mediated genomic plasticity and diversity among potential effector proteins within the genus Coxiella. *Infect Immun* 77(2), 642-656. doi: 10.1128/iai.01141-08.

Coleman, S.A., Fischer, E.R., Howe, D., Mead, D.J., and Heinzen, R.A. (2004). Temporal analysis of Coxiella burnetii morphological differentiation. *J Bacteriol* 186(21), 7344-7352. doi: 10.1128/jb.186.21.7344-7352.2004.

Seshadri, R., Paulsen, I.T., Eisen, J.A., Read, T.D., Nelson, K.E., Nelson, W.C., et al. (2003). Complete genome sequence of the Q-fever pathogen Coxiella burnetii. *Proc Natl Acad Sci U S A* 100(9), 5455-5460. doi: 10.1073/pnas.0931379100.

Walter, M.C., Vincent, G.A., Stenos, J., Graves, S., and Frangoulidis, D. (2014). Genome Sequence of Coxiella burnetii Strain AuQ01 (Arandale) from an Australian Patient with Acute Q Fever. *Genome Announc* 2(5). doi: 10.1128/genomeA.00964-14.
